# Molecular Signature of Indeterminate Thyroid Lesions: Current Methods to Improve Fine Needle Aspiration Cytology (FNAC) Diagnosis

**DOI:** 10.3390/ijms18040775

**Published:** 2017-04-06

**Authors:** Silvia Cantara, Carlotta Marzocchi, Tania Pilli, Sandro Cardinale, Raffaella Forleo, Maria Grazia Castagna, Furio Pacini

**Affiliations:** Department of Medical, Surgical and Neurological Sciences, University of Siena, 53100 Siena, Italy; carlottamarzocchi@libero.it (C.M.); t.pilli.e@ao-siena.toscana.it (T.P.); sandro.cardinale@gmail.com (S.C.); forleo.r@gmail.com (R.F.); m.g.castagna@ao-siena.toscana.it (M.G.C.); furio.pacini@unisi.it (F.P.)

**Keywords:** fine needle aspiration cytology (FNAC), indeterminate lesions, next generation sequencing, gene expression classifier, microRNAs (miRNAs)

## Abstract

Fine needle aspiration cytology (FNAC) represents the gold standard for determining the nature of thyroid nodules. It is a reliable method with good sensitivity and specificity. However, indeterminate lesions remain a diagnostic challenge and researchers have contributed molecular markers to search for in cytological material to refine FNAC diagnosis and avoid unnecessary surgeries. Nowadays, several “home-made” methods as well as commercial tests are available to investigate the molecular signature of an aspirate. Moreover, other markers (i.e., microRNA, and circulating tumor cells) have been proposed to discriminate benign from malignant thyroid lesions. Here, we review the literature and provide data from our laboratory on mutational analysis of FNAC material and circulating microRNA expression obtained in the last 6 years.

## 1. Thyroid Nodules

In countries where iodine deficiency has been corrected by iodine prophylaxis, thyroid nodules are found in approximately 4–7% of the population [1]. However, in countries affected by moderate or severe iodine deficiency, the prevalence is even greater [2]. Subclinical nodules, detected by thyroid ultrasound, are found in over 50% of women older than 60 years, a number similar to that reported in autopsy series.

Any type of nodule may be found as a single lump in an otherwise normal thyroid gland or in the context of a multinodular goitre. Regardless of the presentation, the large majority are benign hyperplastic nodules, frequently an expression of underlying nodular goitre or autoimmune thyroiditis. Thyroid cancer is found in less than 10% of hypo-functioning nodules that are solid or mixed on thyroid ultrasound (US) and more than 80% of them are differentiated thyroid cancer of the follicular epithelium.

Surgical treatment of thyroid nodules without selection would expose millions of people annually to surgery. Since only a small proportion of these nodules finally result malignant at histology, this approach would imply a tremendous number of unnecessary surgeries and high financial costs. Thyroid nodules must therefore undergo rigorous selection based on a rational diagnostic protocol.

## 2. Diagnostic Evaluation of Thyroid Nodules: Fine-Needle Aspiration Cytology (FNAC)

The ultimate objective of the diagnostic protocol is to differentiate between benign and malignant nodules. Nowadays, the problem has largely been solved by fine-needle aspirate cytology. In expert hands, FNAC has an overall accuracy of 95%. The sensitivity is between 43% and 98% and the specificity is between 72% and 100%, with positive and negative predictive values of 89–98% and 94–99%, respectively [3]. False positive and false negative results are between 1–11% and 0–7%, respectively.

The Bethesda Classification System [4], a six diagnostic category system, is at present the most widespread reporting system for thyroid fine needle aspiration (FNA) cytology. The categories are: (I) non-diagnostic/unsatisfactory; (II) benign; (III) atypia of undetermined significance/follicular lesion of undetermined significance (AUS/FLUS); (IV) follicular neoplasm/suspicious for follicular neoplasm (FN); (V) suspicious for malignancy (SUSP) and (VI) malignant.

Category I refers to samples where inadequate or insufficient material is present for a diagnosis or the interpretation is precluded by technical artefacts. In different series, the rate of inadequate cytologies varies between 15% and 20% and in these cases it is recommended to repeat the procedure after some weeks or months [5,6,7,8]. Around 70% (range 53–90%) of aspirates are classified as category II, meaning that the features are consistent with a nodular goitre or thyroiditis. Meanwhile 4% (1–10%) are classified as category VI when unequivocal features of papillary, medullary or anaplastic carcinoma or lymphoma are present, and 10% (5–23%) are classified as category V. A particular issue is represented by category III and IV, representing nearly 20% of FNACs. In this case, the aspirate is represented by a monotonous population of follicular cells arranged on cohesive groups, whose cellular and nuclear features are similar whether the nodule is benign or malignant. The distinction is based on the presence of vascular and capsular invasion, which is detectable only at histology. In a meta-analysis of 25,445 thyroid FNAC [4] cases reported from eight studies using the Bethesda System, 9.6% of all samples were diagnosed as AUS/FLUS (category III) and 10.1% were diagnosed as follicular neoplasm/suspicious for follicular neoplasm (FN/SFN) (category IV) with an average cancer risk at final histology of 15.9% and 26.1%, respectively. It is evident that, both the AUS/FLUS and FN/SFN have a cancer risk that cannot be ignored. However, at final histology, only about 25% of the lesions result malignant, so the risk of cancer is not high enough to definitely support surgery as treatment of all indeterminate lesions.

## 3. Protein-Based Assays to Increase FNAC Performance

To avoid unnecessary surgeries and to increase FNAC performance especially for Categories III and IV, several markers have been proposed in the past years. Among those studied by immunocytochemistry, galectin-3 is one of the most reliable. Galectins are carbohydrate-binding proteins that are members of the β-galactoside binding lectin family. Galectin-3, (Gal-3) appears to be necessary for the maintenance of transformed thyroid papillary cancer (PTC) cell lines in vitro [9]. The use of Gal-3 in the detection of thyroid malignancy in indeterminate or suspicious FNA has a sensitivity that ranges from 20% to 100% and a specificity ranging from 62% to 100% [10,11,12,13,14,15,16,17]. In case of indeterminate FNAC with a positive staining for Gal-3, surgery is strongly recommended, however no specific suggestions can be made in case of Gal-3 negative staining [14]. Similar results have been described with the Hector Battiflora Mesothelial-1 (HBME-1), a monoclonal antibody developed against the microvillous surface of mesothelial cells, which has shown a sensitivity of 79–87% and a specificity of 83–96% [12,13,18,19,20] in Bethesda categories III and IV. Another proposed marker is CD44v6, a polymorphic family of immunologically related cell-surface glycoproteins, which have a functional role in regulating cell–cell and cell–matrix interactions, cell migration, tumor growth and progression [21,22,23,24]. The combined use of CD44v6 and Galectin-3 in indeterminate lesions showed 88% sensitivity, 98% specificity with a positive predictive value (PPV) of 91%, and a diagnostic accuracy of 97% [15].

Although the usage of different markers has been tested to improve the diagnostic efficacy in FNAC, so far none of the tested molecules has provided sufficient sensitivity and specificity to advocate its use in routine practice (not recommended by the America Thyroid Association (ATA).

## 4. Use of Molecular Markers in the Differential Diagnosis of Thyroid Nodules

The discovery of genetic alterations specific for differentiated thyroid cancer have provided molecular markers to be searched for in the material obtained by FNA, thus increasing the diagnostic accuracy of traditional cytology. The need to search for genetic alterations in FNAC sample should be considered especially for Bethesda categories III and IV. However, the revised guidelines for the management of thyroid cancer published by ATA in 2015 [25] do not provide strong recommendation in support of the use of molecular markers to help the management of patients with indeterminate cytology.

The most frequent genetic alterations detected in papillary and follicular thyroid carcinoma (PTC and FTC, respectively) are B-Raf proto-oncogene, serine/threonine kinase (BRAF), rat sarcoma (N-H-KRAS) point mutations, and REarranged during Transfection proto-oncogene (RET)/PTC, and Paired box 8/Peroxisome proliferator activated receptor gamma (PAX8/PPARγ) rearrangements [26,27]. Telomerase reverse transcriptase (TERT) promoter and Tumor protein 53 (TP53) mutations are more frequent in less differentiated carcinomas [28,29,30]. Nowadays, there are three diffuse approaches to investigating the molecular profile of FNA: (1) the seven genes panel; (2) the Afirma classifier and (3) next generation sequencing (NGS) assays.

### 4.1. Seven Genes Mutational Panel

The first study analyzing the contribution of molecular testing to thyroid fine-needle aspiration cytology was published in 2010 [31]. In this work, the authors considered BRAF and RAS gene mutations, as well as RET/PTC, and PAX8/PPAR-γ gene rearrangements in 117 indeterminate cytologies. Among these, 35 (29.9%) cases had a neoplastic outcome and 20 (17.1%) cases were found to be carcinoma. Positive molecular results were found in 12 cases, all of which were PTC. The authors found that the cancer probability for AUS/FLUS and FN/SFN with molecular alteration was 100%, while the probability for AUS/FLUS and FN/SFN without molecular alteration was 7.6%.

In the same year, another study [32] analyzed 174 consecutive FNAC (all categories) for BRAF, RAS, RET, TRK, and PPAR-γ alterations. Mutations were found in 67/235 (28.5%) cytological samples. Of the 67 mutated samples, 23 (34.3%) were mutated by RAS, 33 (49.3%) by BRAF, and 11 (16.4%) by RET/PTC. The presence of mutations at cytology was associated with cancer in 91.1% of the cases and with follicular adenoma in 8.9% of the time. The accuracy of molecular analysis was 90.2%, with a sensitivity of 78.2%, specificity of 96.2%, PPV of 91% and negative predictive value (NPV) of 89.9%. Considering only categories III and IV (*n* = 41), the authors found that 7/41 (17%) samples were mutated (2 BRAF, 2 RET-PTC, 3 RAS). At final histology, all but one (follicular adenoma) were PTC. Of the 34 samples with no mutation, 33 were benign lesions and only one was PTC. Specificity was 97%, sensitivity was 85% and accuracy 95%.

The most complete work aimed to disclose the clinical utility of molecular testing of thyroid FNA samples with indeterminate cytology was published in 2011 [33]. Nikiforov and co-workers analyzed the presence of BRAF, N-H and K-RAS point mutations and RET/PTC1-3, PAX8/PPARγ rearrangements in 1056 consecutive thyroid FNA samples with indeterminate cytology. In 967/1056 (92%) cytologies, the material was adequate for molecular analysis. They found 87 mutations including 62 RAS (71.3%), 19 BRAF (21.8%), 1 RET/PTC (1.1%) and 5 PAX8/PPARγ rearrangements (5.8%). In the AUS/FLUS category, sensitivity was 63%, specificity 99%, PPV 88%, NPV 94% and accuracy 94%. For the FN/SFN group, sensitivity was 57%, specificity 97%, PPV 87%, NPV 86% and accuracy 86%. In AUS/FLUS, FN/SFN categories the detection of any mutation conferred the risk of histological malignancy of 88 and 87%, respectively. The risk of cancer in mutation-negative nodules was 6%, 14%, and 28%, respectively.

In conclusion, mutation panels intended to identify malignancies in indeterminate lesions must include at least BRAF and RAS point mutations (H, K and NRAS), and RET/PTC, PAX8/PPAR-γ rearrangements. Several “homemade” methods comprising PCR with final Sanger sequencing and some commercial kits are available to screen for these alterations with the limitation that they cannot rule out malignancy with a NPV > 95%.

Since the publication of our previous work [32], we applied molecular testing in clinical routine, especially for FNAC categories III and IV. We collected 197 consecutive indeterminate samples and searched for BRAF, RAS (H, K and NRAS), and TERT point mutations, and RET/PTC1-3 and PAX8/PPAR-γ rearrangements. End point PCR, real time PCR, denaturing high performance liquid chromatography (DHPLC) and direct sequencing were used for the analysis [32]. The exam was performed on 176/197 (89.4%) of the sample as in 21/197 (10.6%) the collected material was inadequate for the investigation. We found 17 mutations (9.6%) including 3 BRAF, 2 HRAS, 5 NRAS, 1 KRAS and 6 RET/PTCs. These 17 patients were subjected to surgery and 15/17 (88.2%) were confirmed malignant at final histology (3 FTC, 5 PTC and 7 follicular variant PTC) whereas 2/17 (11.7%) were follicular adenoma (1 NRAS and 1 RET/PTC). Among the 159 nodules negative for mutations, 23 underwent surgery for other reasons (i.e., ultrasound characteristics, patient's decision, increased nodule size over time) and 21/23 (91.3%) were confirmed benign lesions at histology whereas 2/23 (8.6%) were malignant (2 microcarcinomas). The PPV was 88.2% and the NPV was 91.3%, with an accuracy of 90% (Table 1). One-hundred and thirty-six nodules/176 (77.2%) negative for mutation and not subjected to surgery are still under follow up. In a period of time from 1 up to 6 years, no increase in nodule size or changes in ultrasound features were observed. Twenty-two/136 (16.2%) samples repeated a second FNAC and a category II was found for these lesions confirming the results of molecular test. Despite the encouraging results, the method of the “seven genes” has the limitation that collected material can be inadequate to perform the complete panel, thus increasing the number of false negative results.

### 4.2. Afirma Classifier

The Afirma test is a gene expression classifier (GEC) [34] which uses the expression of 142 genes to categorize thyroid nodules into benign or suspicious (rule out method). The test was validated in a multi-institutional (for a total of 49 clinical sites) prospective double-blind study funded by industry (Veracyte) in indeterminate nodules [35]. Authors obtained 577 cytologically-indeterminate aspirates, 413 of which had corresponding histopathological specimens from excised lesions. After inclusion criteria were met, only 265 aspirated were allocated to GEC and were included in the final analysis [35]. Of these 265, 85 (32%) were confirmed to be malignant at histology. In the 265 indeterminate cytology nodules, the sensitivity of the Afirma test was 92% (95% confidence interval (CI), 84%, 97%, 78/85) and the specificity was 52% (95% CI, 44%, 59%, 93/180). In another study by same authors [36] on 339 cytologically-indeterminate nodules (165 AUS/FLUS; 161 FN; 13 suspicious for malignancy), 174/339 (51%) were GEC benign and 148/339 (44%) were GEC suspicious. Among GEC-suspicious nodules, 121 were surgically removed and 53 (44%) were malignant, confirming the previous study in terms of sensitivity and specificity. Recent studies, have shown results from the GEC classifier in indeterminate cytologies obtaining high sensitivity but lower specificity compared to previous reports thus stressing the need of additional, independent, non-industry supported studies to establish the performance of the classifier [37,38,39]. In summary, based on the above studies, Afirma test sensitivity has been reported to range from 83% to 100% and specificity from 7 to 52%, where the prevalence of malignancy in histopathologically confirmed study populations has ranged from 17% to 51% [35,37,38,40].

### 4.3. Thyroseq and Other NGS Platform

Targeted next generation sequencing (NGS) is a promising method to simultaneously examine multiple genes with high sensitivity potentially achieving not only high PPV but also high negative predictive value (NPV) [41] and with low input of starting material (5 to 10 ng).

ThyroSeq is a NGS-based gene mutation and fusion panel initially designed to target 12 cancer genes with 284 mutational hot spots [41], showing 100% accuracy with a sensitivity of 3–5% of mutant alleles. In the first work reporting data from Thyroseq, the authors analyzed 229 thyroid neoplastic and non-neoplastic samples and found mutation in 70% of PTCs (19/27), 83% of papillary thyroid cancer follicular variant (PTCFV) (25/30), 59% of FTC (21/36), 30% of poorly differentiated thyroid carcinoma (3/10), 74% of anaplastic thyroid cancer (ATC) (20/27) and in 73% of medullary thyroid carcinomas (11/15). The majority of samples were mutated for BRAF and RAS. Other studies confirmed the high PPV of the ThyroSeq (88% and 87% for AUS/FLUS and FN, respectively) [42,43] and indicate that the test could potentially be used as a “rule in” test.

In 2014, results from ThyroSeq v2, an enhanced version of the test, on AUS/FLUS and FN cytologies were published [44]. ThyroSeq v2 allowed the analysis of 14 genes (more than 1000 mutations) and RNA alterations (approximately 42 fusions) reaching a sensitivity and specificity of 90% and 93%, respectively, a PPV of 83%, an NPV of 96%, and accuracy of 92% [44]. These results suggested that ThyroSeq v2 may potentially works as both “rule out” and “rule in” test for nodules with indeterminate cytology. Finally, owing to the limited studies and data from literature, the value of ThyroSeq v2 needs further investigation. Moreover, clinical validation results are not available yet, whereas data for lung and other tumors show that next generation sequencing is as robust as Sanger sequencing in routine diagnostics and, in addition, is able to reveal mutations in low percentage and screen the mutational status of different critical samples offering innovative diagnostic opportunities [45,46,47,48]. Furthermore, methodological problems like result interpretation (e.g., for unknown mutations), definition of cut offs for mutation calling, and bioinformatics analysis need to be solved and common standard operation procedures (SOPs) need to be defined. On the matter of bioinformatics analysis, a recent report describes a solution called “SeqReport” [49]. This module automatically imports patient data and related NGS run information and allows comprehensive review of all variants by users linking to both COSMIC and dbSNP databases and manual review of variants. In addition, the program automatically locates variants with low frequency or coverage and compares the status of Sanger sequencing confirmation. In this method, the cut off values are determined for each multigene panel during validation. Human errors are minimized and the creation of clinical report is automatic also with appropriate clinical comments.

Le Mercier and colleagues [50] performed a pilot study with a commercially available NGS-based 50-gene panel kit (Ion AmpliSeq Cancer Hotspot Panel version 2; Thermo Fisher Scientific, Gent, Belgium) to evaluate 34 indeterminate FNA samples. The panel is designed to amplify 207 amplicons covering approximately 2800 COSMIC mutations from 50 oncogenes and tumor suppressor genes. The authors identified cytologies with a “molecular test negative” (including patients carrying germline polymorphisms, mutations of unknown clinical significance, or no mutation) or “molecular test positive” for patients carrying pathogenic mutations reaching a sensitivity and specificity of 71% and 89%, respectively. The PPV and NPV were 63% and 92%, respectively, with an accuracy of 85%. ThyroSeq v2 actually has shown the best results in terms of sensitivity, specificity, PPV and NPV but further studies including a larger number of cases are required for the Ion AmpliSeq Panel.

## 5. Role of miRNAs in the Differential Diagnosis of Thyroid Lesions

MicroRNA (miRNAs) are small molecules of RNA (approximately 22 nt), non-encoding for protein which negatively regulate gene expression-targeting specific mRNAs [51]. miRNAs can be detected in plasma and serum as they circulate in the blood in a stable, cell-free form [52]. Furthermore, tumor cells have been shown to release miRNAs into the circulation [52] and profiles of miRNAs in plasma and serum have been found to be altered in cancer and other disease states [53,54,55]. Larger scale miRNA analysis has proven that miRNA expression enables the distinction of benign tissues from their malignant counterparts [56,57]. The mechanisms of miRNA implication in cancer development are linked to downregulation of tumor suppressor genes or upregulation of oncogenes.

Several studies have demonstrated a different miRNA signature between benign and malignant thyroid tissues [58,59,60,61,62,63,64,65] unfortunately using different detection systems (microarray and/or Quantative RT-PCR (Q-RT-PCR) and producing inconsistent results in terms of selected miRNAs, sensitivity and specificity. However, all authors concluded that a limited set of miRNAs can be used for the differential diagnosis between benign and malignant lesions in the surgical samples with high accuracy, implying the potential role of miRNAs in differentiating the nature of thyroid nodules in FNAC. Again, the most important question is question is whether the analysis of miRNAs in cytological samples can improve FNAC results, particularly for indeterminate lesions [61,66,67,68,69,70,71,72,73,74]. All the studies which addressed this issue obtained a similar diagnostic odds ratio (mean 20.3) and concluded that a set of multiple miRNAs seems to be more sensitive (sensitivity of 87%) than a single miRNA (sensitivity of 71%) although there is discrepancy in terms of set of miRNA proposed. Pooling together the results from these studies, however, a relative small set of 15 miRNAs emerge as the more powerful diagnostic panel for indeterminate lesions. The panel is composed of miRNA7, -146, -146b, -155, -221, -222, -21, -31, -187, -30a-3p, -30d, -146b-5p, -199b-5p, -328 and miRNA197. Future prospective and retrospective research are recommended on a large cohort of indeterminate lesions to validate the diagnostic value of this panel.

As pointed out previously, FNAC represents the gold standard for the differential diagnosis of thyroid nodules, however it is an invasive technique compared to blood sampling. Thus, the idea is to use miRNAs as a serological marker for thyroid cancer (TC) diagnosis from the moment that TC releases miRNAs into the bloodstream. Three studies addressed this issue [75,76,77], two of them in the Chinese population [77,78] and only one study in the Caucasian population [77]. Although these studies found different set of miRNAs, the preliminary results are promising for future research showing a good sensitivity (ranging from 61.4 to 94%) and specificity (ranging from 57.9% to 98.7%).

We performed a study on serum miRNA expression (miRNA95 and miRNA190) on 982 consecutive patients undergoing FNAC at our institute. We collected serum from 114/982 (11.6%) subjects with a Bethesda III and IV FNAC result. Seventy-five/114 (65.7%) underwent surgery and at final histology we had 11 follicular adenomas (FA), 32 hyperplastic nodules (HN), 27 PTCs, 4 FTCs and 1 Hurthle cell carcinoma (HC). miRNAs were extracted from 200 µL of serum using the miRNeasy Serum/Plasma Kit (Qiagen, Milan, Italy) and retro-transcribed by miScript II RT Kit (Qiagen). Two µL of cDNA was used as template for real time PCR (RT-PCR) to measure miRNA expression levels with the miScript SYBR Green PCR Kit (Qiagen) with specific primers for miRNA-95 and -190 (Qiagen). RT-PCR was performed in duplicate on the Rotor-gene Q MDx (Qiagen) under the following cycling conditions: 95 °C for 15 min, 40 cycles at 94 °C for 15 s, 55 °C for 30 s and 70 °C for 30 s. Relative expression levels were calculated using the 2^−ΔΔ*C*t^ method with the miRNA-16 as endogenous control. miRNA expression correctly identified 38/43 (specificity of 88.4%) of the benign lesions and 27/32 (sensitivity of 84.3%) of malignant sample. We had 5 false positive (FP) (5 HNs) and 5 false negative (FN) (4 PTCs, 1 FTC) results, obtaining a final accuracy of 86.7% (Table 2). Despite the promising results, molecular analysis on FNAC has a better performance and further studies are required to identify the optimal set of circulating miRNAs specific for indeterminate lesions.

Serum normally contains low amounts of total RNA, of which miRNAs only constitute 0.4–0.5%. In addition, serum samples may be affected by technical problems, such as hemolysis and it is not known whether circulating serum expression can be influenced by other comorbidities. In this view, the analysis on FNAC may be preferable.

In 2016 two studies [78,79] were published on clinical validation of the RosettaGX Reveal test, a miRNA-based assay which evaluates a set of 24 miRNAs (by real time PCR) specific for cytologically indeterminate thyroid nodules. The assay can be used directly on FNA smears and it is able to categorize benign or suspicious nodules even when as little as 1% of thyroid cells is present or less than 5 ng RNA are extracted. The overall NPV reported was 99%, with sensitivity of 98% and specificity of 78%.

## 6. Proteomics: An Interesting Alternative Approach to Stratify Thyroid FNAC

Proteomics is the large-scale study of proteins and it is widely used to discover cancer biomarkers. In the field of thyroid cancer, proteomics has been initially applied on thyroid tissue specimens and cancer cell lines [80,81,82,83,84,85,86,87,88,89], using different techniques such as surface-enhanced laser desorption/ionization-time-of-flight-mass spectrometry (SELDI-TOF-MS), liquid chromatography–mass spectrometry (LC/MS) and MS alone. All these studies ended by identifying specific protein signatures for malignant and benign lesions with a final selection of clusters of proteins with discriminating abilities. In particular, proteins involved in oxidative stress, metabolic pathways, nuclear stability, turnover of thyroglobulin, and kinase signaling are those more represented in thyroid cancer. Techniques such as matrix-assisted laser desorption/ionization (MALDI)-TOF-MS and MALDI-imaging mass spectrometry (MALDI-IMS) have been applied in several studies [90,91,92,93,94,95] to cytological thyroid specimens. Most of these studies used ex vivo FNA [90,91,93,94,95] and one study [94] used pre-surgical FNAC obtaining an overall sensitivity of 87% and specificity of 94% in discriminating benign from suspicious samples, with a good reproducibility among studies. Proteomics could serve to improve the preoperative diagnosis of indeterminate lesions, but some aspects such as the limitation in the availability of these technologies and the lack of uniformity among techniques, need to be addressed before its introduction in clinical practice.

## 7. Conclusions

In summary, the purpose of thyroid molecular testing is to discriminate the nature of thyroid nodules and reduce the diagnostic uncertainty of cytologically indeterminate lesions prior to surgery. Mutation panels intended to identify malignancies must include at least BRAF, and RAS point mutations as well as RET/PTC, NTRK, and PAX8/PPARγ rearrangements. Several “home-made” methods and some commercial kits are available to screen for these alterations with the limitation that they cannot rule out malignancy with an NPV >95%. GEC recognizes benign lesions on the basis of an expression pattern of mRNA extracted from one or two dedicated FNA needle passes. A negative result in the Afirma test has resulted in a major decrease in the number of surgeries performed in samples classified as Bethesda categories III and IV. However, Afirma shows a low PPV. On the other hand, the risk of malignancy calculated by ThyroSeq or other NGS platforms is superior to that of the Afirma, reaching an NPV of 95% or more, with good sensitivity and high PPV. The identification of new biomarkers (i.e., miRNA, proteomic profiles) in the thyroid needs to be corroborated in larger studies with final histology as a gold standard and adequate follow up before use in the clinical routine [96]. Therefore, molecular testing must be always performed in specialized laboratories and results interpreted within the context of the clinical, radiographic, and cytological findings. In addition, clinicians may take into account that the interpretation of molecular testing and its utility are strongly influenced by the prevalence of cancer in each cytological category [96] which can differ among centers. Due to this aspect, molecular test performance may vary significantly.

## Figures and Tables

**Table 1:** Results from mutation analysis on indeterminate lesions treated with surgery.

Atypia of Undetermined Significance/Follicular Lesions of Undetermined Significance (AUS/FLUS)			
Follicular neoplasma/suspicious for follicular neoplasma (FN/SFN)			
(*n* = 40)			
	**Histology Malignant**	**Histology Benign**	Sensitivity 88.2% Specificity 91.3% PPV 88.2% NPV 91.3% Accuracy 90%
Mutation positive (*n* = 17)	7 RAS (6 FVPTC, 1 FTC) 3 BRAF (3 PTC) 5 RET/PTC (1 FVPTC, 2 PTC, 2 FTC)	1 NRAS (FA) 1 RET/PTC (FA)	
Mutation negative (*n* = 23)	2 microcarcinoma	21 (9 FA, 12 HN)	

PTC = papillary thyroid cancer; FTC = follicular thyroid cancer; FVPTC = follicular variant of PTC; FA = follicular adenoma; HN = hyperplastic nodules; PPV = positive predictive value; NPV = negative predictive value.

**Table 2:** Results from microRNA (miRNA) expression analysis on indeterminate lesions treated with surgery.

Histology	miRNA Expression Negative for Malignancy	miRNA Expression Positive for Malignancy	Performance
Benign at histology	38 (11 FA, 27 HN)	5 (5 HN)	Sensitivity 84.3% Specificity 88.4% PPV 88.4% NPV 84.3% Accuracy 86.7%
Malignant at histology	5 (1 FTC, 4 PTC)	27 (23 PTC, 3 FTC, 1 HC)	

PTC = papillary thyroid cancer, FTC = follicular thyroid cancer, FA = follicular adenoma, HN = hyperplastic nodules, HC = Hurtlhe cell carcinoma, PPV = positive predictive value, NPV = negative predictive value.
